# Genetic Structures of *Crassostrea ariakensis* Generations Based on Partial Mitochondrial *cox1* and *rrnL* Indicated a High Breeding Potential After Four-Years Successive Selections

**DOI:** 10.3390/ani16030451

**Published:** 2026-02-01

**Authors:** Ming Yan, Peizhen Ma, Zhihong Liu, Zhuanzhuan Li, Xianglun Li, Tao Yu, Weijun Wang, Chengwu Wang, Xiujun Sun, Liqing Zhou, Biao Wu

**Affiliations:** 1 National Demonstration Center for Experimental Fisheries Science Education, Shanghai Ocean University, Shanghai 201306, China; 19083642536@163.com (M.Y.); 13165264134@163.com (X.L.); 2 State Key Laboratory of Mariculture Biobreeding and Sustainable Goods, Yellow Sea Fisheries Research Institute, Chinese Academy of Fishery Sciences, Qingdao 266071, China; mapz@ysfri.ac.cn (P.M.); liuzh@ysfri.ac.cn (Z.L.); lizz@ysfri.ac.cn (Z.L.); xjsun@ysfri.ac.cn (X.S.); zhoulq@ysfri.ac.cn (L.Z.); 3 Laboratory for Marine Fisheries Science and Food Production Processes, Qingdao Marine Science and Technology Center, Qingdao 266237, China; 4 Changdao Enhancement and Experiment Station, Chinese Academy of Fishery Sciences, Yantai 265800, China; cdyutao@126.com; 5 School of Agriculture, Ludong University, Yantai 264025, China; wwj2530616@163.com; 6 Yantai Beizhiyuan Biotechnology Co., Ltd., Yantai 261400, China; ytwangchengwu@163.com

**Keywords:** oyster, selected breeding, genetic diversity, population, aquaculture

## Abstract

With the rapid development of oyster farming and the emergence of new varieties, the identification and evaluation of genetic resources have become fundamental to the effective utilization of oyster germplasm resources. To evaluate the impact of selective breeding on the germplasm of *Crassostrea ariakensis*, this study conducted genetic structure analysis on five successive generations including four generations selected (F1 to F4) focusing on both fast growth and high glycogen content and one base population (F0) based on partial mitochondrial *cox1* and *rrnL* gene sequences. Results showed that the 649 bp *cox1* and 488 bp *rrnL* sequences exhibited (A+T)-biased composition and showed positive AT skew. A total of 12 haplotypes were found for the *cox1* sequences, while only three haplotypes were detected for the *rrnL* sequences. However, among the F1 to F4 populations, only three *cox1* haplotypes and two *rrnL* haplotypes were observed. Based on *cox1* sequences, the values of the haplotype diversity index, average number of nucleotide differences, and nucleotide diversity index all decreased progressively from F1 to F4, indicating a reduction in genetic variation due to selective breeding. All populations exhibited a low level of nucleotide diversity (<0.05). In addition, the intra-population genetic distance declined from F0 to F4, with significant genetic differentiation between F0 and F1, as well as between F0 and F4 (*p* < 0.05). In contrast, only the F0 and F4 population had multiple *rrnL* haplotypes, and no statistically significant genetic differentiations were observed based on *rrnL* fragments (*p* > 0.05). The AMOVA results showed that intra-population genetic variation exceeded inter-population variation, regardless of whether it was assessed using the *cox1* gene or the *rrnL* gene, indicating that substantial genetic diversity persists within populations despite multiple generations of selection, and complete genetic differentiation across generations has not been achieved. These findings indicate that the selectively bred lines retain considerable genetic potential and can serve as a valuable resource for future breeding programs.

## Simple Summary

The genetic diversities of five successive generations, including the base population (F0) and four selective breeding populations (F1 to F4) of *Crassostrea ariakensis*, were analyzed based on both mitochondrial *cox1* and *rrnL* gene sequence fragments. The *cox1* gene sequences exhibited higher sequence variability compared to the *rrnL*. Based on the partial *cox1* sequences, the genetic diversity indices, such as the haplotype diversity, average number of nucleotide differences, and nucleotide diversity, as well as the intra-population genetic distances, showed a decline across generations from F0 to F4, indicating a reduction in genetic variation due to selective breeding. Although the generations became more genetically conserved and very low genetic differentiation occurred as the selective breeding lasted, the F4 population still maintained relatively high levels of genetic diversity. These findings suggest that substantial within-populations variation persists even after multiple rounds of selection, and complete genetic homogenization across generations has not been achieved.

## 1. Introduction

As the most extensively farmed shellfish worldwide [1], oysters support a massive aquaculture industry, with production in China alone exceeding 7.25 million tons in 2024 [2]. Given this scale, the future healthy development of the industry therefore depends not only on increasing production but, crucially, on breeding new varieties with superior traits [3]. Along with the application of breeding techniques, such as selective breeding [4,5], crossbreeding [6,7], and polyploidy breeding [8,9], genetic improvements have emerged as a key research focus, leading to the development of numerous oyster varieties [10,11,12]. As a foundational resource for genetic improvement, genetic diversity reflects hereditary variation both within and between populations, thereby providing critical guidance for breeding strategies [13].

The population genetic analysis of oysters has advanced significantly due to the development in molecular biology techniques, enabling the use of diverse molecular markers such as gene fragments, randomly amplified polymorphic DNA (RAPD), microsatellite (simple sequence repeats, SSR), restrictive fragment length polymorphism (RFLP), single nucleotide polymorphism (SNP), copy number variation (CNV), and insert missing (InDel, Insertion/Deletion), among others. Based on the mitochondrial *cox1* gene sequences, Li et al. analyzed the genetic diversity and population differentiation of 11 wild groups of *Crassostrea hongkongensis* along the southern coast of China [14]; Li investigated the genetic structures of wild populations of *Crassostrea gigas*, *Crassostrea angulata*, *Crassostrea ariakensis*, and *C. hongkongensis* in China [15]; and Li et al. analyzed the population genetic structure and historical dynamics of *Saccostrea echinata* [16]. In addition, SSRs and SNPs have become widely used markers in the genetic research of oyster populations. Launey et al. utilized microsatellite polymorphism to assess genetic differentiation in *Ostrea edulis* across the European coastline [17]; Li et al. [18], Yu et al. [19], Bai [20], and Kong et al. [21] developed gSSR, EST-SSR and SNP markers for *C. gigas*; Wang and Guo developed and screened 53 polymorphic EST-SSR loci in *Crassostrea virginica* [22]; Li et al. developed the SSR markers for *C. hongkongensis* and analyzed its population genetic structure along the southern coast of China [14]. Ma et al. further developed trinucleotide repeat SSR markers of *C. hongkongensis* [23]; Xiao et al. used eight polymorphic microsatellite markers to investigate the population genetic status of the East Asian *C. ariakensis* population [24]; White et al. identified 10,363 SNP sites of *Ostrea lurida* using whole-genome resequencing data [25]; Wu et al. [26] resequenced 261 individuals from the five geographical populations of *C. ariakensis* and identified 1,927,746 high-quality SNP markers; Mao et al. used whole-genome SNPs and analyzed the genetic diversity and breeding characteristics of *C. gigas* in Dalian [27].

Among the breeding populations, Zhang et al. found that the selected fast-growing population of *C. gigas* exhibited high genetic diversity based on SSR markers [28]; Zhong used EST-SNP markers to compare genetic diversity between wild populations and six breeding generations of *C. gigas* with rapid growth, revealing a decreasing trend in genetic diversity of breeding populations [29]; Guo et al. used eight SSR markers and found that successive breeding had a certain impact on the genetic differentiation of *C. angulata* populations [30]; Zhang et al. integrated SSR and *cox1* sequence data to compare the population genetics of four shell color strains of *C. gigas,* proposing that the genetic structures of these strains had become fully differentiated [31]; Li et al. used 21 microsatellite primers to analyze the genetic structures of two selected rapid-growth populations (focusing on shell length and height respectively) and wild populations of *C. gigas*, finding that all populations had high genetic diversity and low genetic differentiation [32]; Xuan et al. analyzed *cox1* and mitochondrial non-coding region (MNR) sequences of *Crassostrea sikamea* among cultured populations in the USA and wild populations in China and Japan, suggesting that wild populations in China possessed higher genetic diversity than those in Japan and the USA [33].

In addition to population genetic analysis, molecular tools can also promote the production of shellfish. Through genome-wide association analysis (GWAS) and molecular marker assisted selection (MAS), breeders can significantly improve the breeding efficiency of target traits. For example, GWAS provides valuable information for the genetic improvement of low salt tolerance in *C. gigas* [34]. Genome wide association analysis is used to screen for a subset of SNPs significantly associated with the target trait, rather than using all markers, which significantly reduces typing costs while ensuring prediction accuracy [35]. Environmental adaptability improvement is equally important, and genetic tools help cultivate strains that are tolerant to extreme conditions. Recent studies such as by Díaz-Puente et al. have further validated the value of genetic decoupling and genotype environment interaction in optimizing the production in *Mytilus galloprovincialis* [36,37].

*C. ariakensis* is naturally distributed in estuarine areas and possesses significant economic and ecological potential [38], making it an important species for breeding programs [39,40,41]. As fast growth and high glycogen content are two important economic properties for oyster products [32,42], we conducted the successive selection of *C. ariakensis* targeting both properties. However, the genetic structures of these breeding generations have not yet been reported. In this study, the genetic diversities of the four successive generations of *C. ariakensis* were analyzed using two common genetic markers [18,19,20,43], i.e., mitochondrial *cox1* and *rrnL* genes. The objectives were to elucidate the genetic structure of artificially bred *C. ariakensis* and to evaluate the impact of selective breeding on its germplasm during the breeding process.

## 2. Materials and Methods

### 2.1. Sample Collection

**Figure 1:**
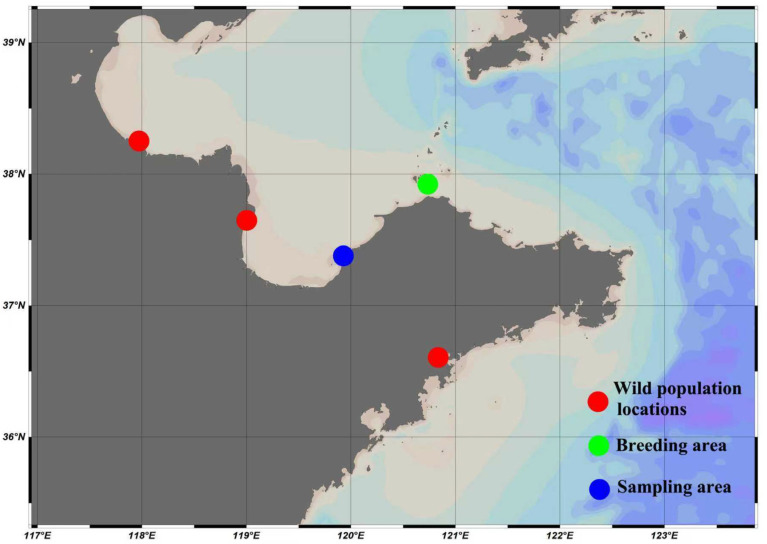
The wild population locations, breeding area and sampling area of *C. ariakensis* in this study.

In 2019, wild *C. ariakensis* oysters were collected from Dongying, Binzhou, and Laiyang in Shandong Province (Figure 1) to constitute a mixed cohort. This cohort was used to establish the basic population (F0) through intervarietal free crossing in 2020. Subsequently, four consecutive rounds of family-based selection were carried out from 2021 to 2024 using one-year-old oysters at the Changdao Enhancement and Experiment Station, Chinese Academy of Fishery Sciences (Figure 1), targeting both fast growth and high glycogen content. Truncation selection was conducted for the parental oysters of each generation [44]. The near-infrared model was used in determining the glycogen contents when selecting parental oysters [45]. *C. ariakensis* was cultivated in seawater ponds at Yantai Beizhiyuan Biotechnology Co., Ltd. in Laizhou, Shandong Province (Figure 1). Thirty-five individuals from each of the four selected generations (F1, F2, F3, and F4) and F0 were randomly sampled for molecular analysis. The adductor muscle was chosen for DNA extraction because of the lower impurity content compared with other tissues. All adductor muscles were collected, preserved and stored in 95% ethanol.

### 2.2. DNA Extraction, Amplification and Sequencing

Genomic DNA was extracted from each oyster using the phenol-chloroform method [46]. The DNA concentration and purity were assessed using an ultra-micro spectrophotometer (NanoDrop 2000, Thermo Fisher Scientific, Waltham, MA, USA). Only DNA samples with A260/A280 ratios ranging from 1.8 to 2.2 were selected for PCR amplification, and the concentration was adjusted to 50 ng/μL. Two mitochondrial markers, i.e., *cox1* and *rrnL* gene fragments, were amplified in this study. The *cox1* primers (5′-3′) were LCO1490, GGTCAACAATCATAAAGATTGG and HCO2198: TAAACTTCAGGGTGACCAAAAAATCA [47], while the *rrnL* primers (5′-3′) were *16S* rRNAar, CGCCTGTTTATCAAAAACAT and *16S* rRNAbr: CCGGTCTGAACTCAGATCACGT [48]. All primers were synthesized by Sangon Biotech (Shanghai, China) Co., Ltd. The PCR reaction was conducted using Eppendorf Mastercycler Gradient AG 22331 (Eppendorf AG, Hamburg, Germany) in a 25 μL reaction volume containing 12.5 μL 2×Taq Plus Master Mix II (Dye Plus, P213, Nanjing Vazyme Biotech Co., Ltd., Nanjing, China), 1 μL forward primer (1 nmol), 1 μL reverse primer (1 nmol), 0.5 μL DNA template, and 10 μL ddH_2_O. The PCR amplification protocols were: initial denaturation at 95 °C for 2 min, followed by 35 cycles of denaturation at 95 °C for 1 min, annealing at 51 °C (for *cox1*)/50 °C (for *rrnL*) for 1 min, and extension at 72 °C for 1 min, and finally extension at 72 °C for 5 min. A 1.5% agarose gel was prepared, and the samples were loaded onto the gel for electrophoresis at 130 V for 30 min. Gel bands were visualized using a gel documentation system. The amplified products were sequenced on an Applied Biosystems™ 3730XL DNA Analyzer (Thermo Fisher Scientific, Waltham, MA, USA) by Sangon Biotech (Shanghai, China) Co., Ltd.

### 2.3. Sequence Alignment and Analysis

The *cox1* and *rrnL* sequences were aligned using ClustalW in Mega 11.0.13, respectively, and adapter sequences at both ends were manually removed [49]. AT skew and GC skew values were calculated using the formulae described by Ma et al. [50]. Nucleotide composition for each of the five populations was determined using MEGA 11.0.13. Again, using MEGA 11.0.13, sequences were grouped by population, and the mean pairwise distances between groups were calculated with both transitions and transversions included under the Kimura 2-parameter model. Phylogenetic relationships were then reconstructed separately from the *cox1* and *rrnL* distance matrices using the neighbor-joining method, with rooting on F0 [51]. Then DnaSP 6 software was employed to calculate the number of polymorphic loci (*P*), the number of haplotypes (*H*), the nucleotide diversity index (*Pi*), the haplotype diversity index (*Hd*), and the average number of nucleotide differences (*K*), as well as the locations of polymorphic sites (at synonymous or nonsynonymous sites) [52]. To examine the genealogical relationships among haplotypes, Arlequin v 3.5 [53] was used to determine the haplotype distribution within each population, and PopART 1.7 was applied to construct the haplotype networks based on *cox1* and *rrnL* sequences [54]. The distribution of genetic variation within and among populations was evaluated using the AMOVA analysis method in Arlequinv3.5, as well as the coefficients of genetic differentiation (*Fst*), sums of variances (SSD), and significance levels of differences.

## 3. Results

### 3.1. Gene Sequence Base Composition

A total of 158 *cox1* sequences and 159 *rrnL* sequences were obtained for the five *C. ariakensis* populations, with sequence lengths of 649 bp for *cox1* and 488 bp for *rrnL*. The base contents of C, G, A and T were 20.6%, 18.3%, 37.8% and 23.3% for *cox1*, and 18.0%, 23.6%, 29.5% and 28.9% for *rrnL*, respectively. Both genes exhibited a (A+T)-biased composition of 60.9% in *cox1* and 58.4% in *rrnL*, as well as positive AT skew (0.237 for *cox1* and 0.010 for *rrnL*). The *cox1* sequences showed a negative GC skew of −0.59, while the *rrnL* sequence showed a positive GC skew of 0.135.

### 3.2. Population Genetic Diversity

**Figure 2:**
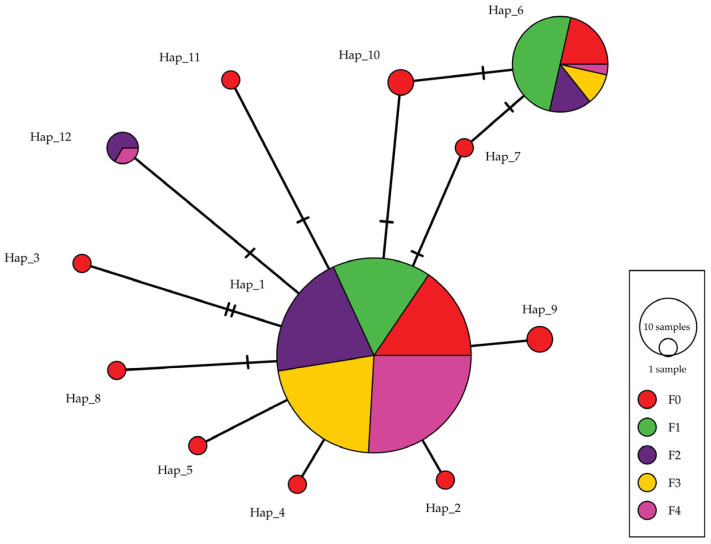
A minimum spanning tree showing the genetic relationships among the 12 *cox1* haplotypes identified in the five *C. ariakensis* populations (F0 to F4).

In total, 12 haplotypes (GenBank accession numbers: PV490271-PV490282) were identified in the *cox1* sequences of *C. ariakensis*, among which Hap_1 had the largest number of individuals (116), accounting for 73.4% of the total, followed by Hap_6 (28 individuals). Hap_1 and Hap_6 were the only haplotypes shared by all five populations. The F0 population harbored 11 haplotypes except Hap_12, which was found in F2 and F4 only. Nine haplotypes, i.e., Hap_2, Hap_3, Hap_4, Hap_5, Hap_7, Hap_8, Hap_9, Hap_10, and Hap_11, were unique to the F0 population (Table 1, Figure 2).

A total of 11 polymorphic loci were identified in the *cox1* sequence across the five populations. Only one polymorphic site (379th base) was located at a nonsynonymous site, changing the Isoleucine to Methionine in two individuals of F0. The F0 population exhibited the highest number of polymorphic loci (*P* = 10), followed by the F2 and F4 population, each with three polymorphic loci. The haplotype diversity values in both the F0 and F1 populations exceeded 0.5, while the F4 population showed the lowest value (0.123). Notably, the values of the haplotype diversity index, average number of nucleotide differences and nucleotide diversity index all decreased progressively through the selective breeding generations from F0 to F4. The average numbers of pairwise differences in the F0 and F1 populations were greater than 1, exceeding those of other populations. All populations showed low nucleotide diversity (<0.05). The highest nucleotide diversity value, observed in F0, was only 0.00185, while that of the F4 population was as low as 0.00029.

**Figure 3:**
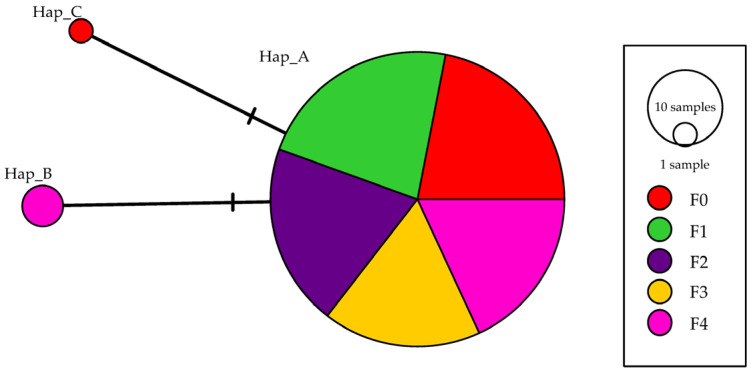
A minimum spanning tree showing the genetic relationships among the three *rrnL* haplotypes identified in the five *C. ariakensis* populations (F0 to F4).

Only three haplotypes (GenBank accession numbers: PV473914-PV473916) were found among the 159 *rrnL* sequences (Figure 3). Among them, Hap_A was shared by 155 individuals across all five populations. Hap_B, found in three individuals, was unique to the F4 population, while Hap_C, represented by a single individual, was unique to the F0 population. Consequently, only the F0 and F4 populations exhibited polymorphic haplotypes. The haplotype diversity indices, mean number of pairwise differences, and nucleotide diversity values for these two populations were 0.714 and 0.123, 1.20336 and 0.18750, and 0.00185 and 0.00029, respectively.

### 3.3. Population Genetic Distance and Differentiation

The genetic distances based on the *cox1* gene fragments for the five *C. ariakensis* populations (Table 2) showed that the F0 population had the highest intra-population genetic distance of 0.00186, followed by the F1 population of 0.00156, while the F4 population exhibited the lowest value of 0.00029. The inter-population genetic distances among the five populations ranged from 0.00046 (between F3 and F4) to 0.00181 (between F0 and F1). Among these, the F0 and F1 populations had relatively high genetic distance values with F2, F3, and F4 populations (>0.001). The genetic differentiation coefficient between populations ranged from −0.02356 (between F2 and F3) to 0.32533 (between F1 and F4). The genetic differentiation between F0 and F1, and between F0 and F4, was significant (*p* < 0.05), while extremely genetic differentiation was found between F1 and three later populations, i.e., F2, F3, and F4 (*p* < 0.01).

**Figure 4:**
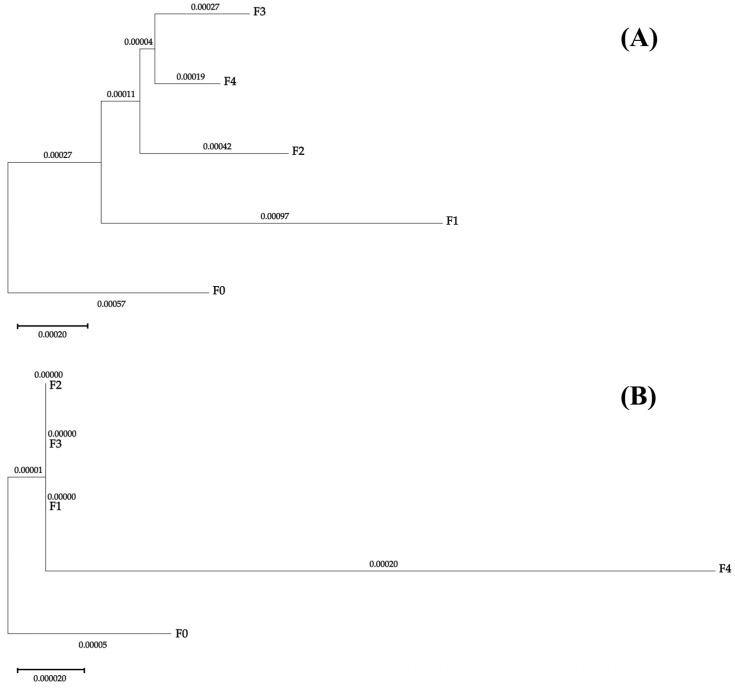
Neighbor-joining tree based on Kimura 2-parameter pairwise distance matrix of partial (**A**) *cox1* and (**B**) *rrnL* genes sequences from the five *C. ariakensis* populations with branch lengths marked.

Since only the F0 population and the F4 population harbored multiple *rrnL* haplotypes, the valid values of genetic distances between populations and within populations, as well as pairwise *Fst*, were much fewer based on partial *rrnL* genes. The intra-population genetic distances based on the *rrnL* gene for F0 and F4 were 0.00012 and 0.00037, respectively (Table 3). The inter-population genetic distance between the F0 and F4 populations was 0.00026, while the genetic distances between F0 and all other populations were 0.00006. The inter-population genetic distances between the F4 population and the other populations (excluding F0 population) were 0.00020. The pairwise *Fst* values between F0 and the other populations ranged from −0.00765 (between F0 and F3) to 0.05490 (between F0 and F4). Furthermore, the populations of F1, F2, and F3 showed closely related generic relationships (Figure 4B). However, the genetic differentiation coefficient values between F4 and all other populations were larger than 0.05. Even so, no statistically significant genetic differentiations were found based on the *rrnL* gene fragments (*p* > 0.05).

The neighbor-joining tree constructed based on the *cox1* gene fragment revealed that the F3 and F4 populations clustered closely, consistent with the genetic distance results (Figure 4A).

The AMOVA results for the *C. ariakensis* populations revealed that genetic differentiation primarily occurred within populations, indicating high levels of intra-population genetic polymorphism. Specifically, based on the *cox1* gene fragments, intra-population genetic variation accounted for 90.20% of the total variation, while inter-population variation contributed 9.80% (Table 4). For the *rrnL* gene, intra-population genetic variation accounted for 94.98%, and inter-population variation accounted for 5.02% (Table 5). The overall genetic differentiation indices for *C. ariakensis* populations were 0.0981 and 0.0502 based on the *cox1* and *rrnL* genes, respectively, indicating very low genetic differentiation among the populations.

## 4. Discussion

### 4.1. Genetic Diversity of the Breeding Generations of C. ariakensis

In this study, the mitochondrial *cox1* and *rrnL* gene sequences of the *C. ariakensis* populations exhibited a nucleotide composition characterized by significantly higher AT content than GC content, consistent with the typical characteristics of invertebrate mitochondrial DNA [55]. Genes with high AT content are more evolutionarily dynamic, demonstrating increased rates of random mutations and genetic drift [56], making them particularly suitable for analyzing intraspecific genetic diversity [57]. Compared to the *rrnL* gene, the *cox1* gene in *C. ariakensis* displayed a greater number of polymorphic sites and haplotypes, as well as higher values of average number of pairwise differences and nucleotide diversity, indicating a higher mutation rate. This pattern has also been observed in other mollusk species, such as *Mytella strigata* [58] and *Donax vittatus* [59].

Due to the wide distribution of *C. ariakensis* and the genetic differentiation between northern and southern populations in China [60], the base population in this study was established using three northern populations. The haplotype diversity of the *cox1* gene fragments in *C. ariakensis* F0 populations was 0.714, with a nucleotide diversity of 0.00185. These values were comparable to those observed in wild oyster populations along China’s coast [18,19,61]. The higher genetic diversity in the F0 population may be attributed to hybridization effects from the parental wild populations, which increased heterozygosity in the offspring [62]. The results indicate that the F0 populations, serving as the base population for selective breeding of *C. ariakensis*, possessed relatively high genetic diversity and provided abundant variation for selecting desirable traits [32], making it an ideal breeding material. The F0 populations exhibited haplotype diversity >0.5 and nucleotide diversity <0.005, consistent with the bottleneck stage of population, during which rapid population growth and mutation accumulation are expected, as proposed by Grant and Bowen [63]. This pattern may be attributed to the recent conservation and restoration effort for oyster reefs in China, which have promoted the recovery of *C. ariakensis* resources [64].

Artificially selected populations are developed by selecting individuals with specific traits to improve or optimize desired characteristics. This selection round process leads to the gradual accumulation or reduction in allele frequencies at loci associated with the target traits, but does not generate new genes [65]. In this study, *C. ariakensis* underwent four successive selections, focusing on both fast growth and high glycogen contents. Although the sample size was limited (28–35 individuals per population), which restricted the accuracy of the results, from a statistical perspective, it was meaningful [14,66,67] and could to some extent reflect the impact of our genetic selection on *C. ariakensis*. Genetic parameters such as haplotype diversity, mean number of nucleotide differences, and nucleotide diversity index gradually decreased across breeding generations. Due to genetic drift and bottleneck effects, artificially selected populations generally exhibited lower genetic diversity compared to wild populations, as observed in the selective breeding of *C. gigas* [33] and *Mimachlamys nobilis* [68]. However, populations with higher genetic diversity are better equipped to adapt to environmental changes, thereby enhancing aquaculture stability and sustainability [69]. Therefore, it is crucial to prevent the decline in genetic diversity during breeding programs to avoid germplasm degradation. In this study, the F4 generation of the *C. ariakensis* population exhibited a haplotype diversity of 0.123 and a nucleotide diversity index of 0.00029 based on the *cox1* gene fragments, which were comparable to values reported for *C. gigas* populations from Zhoushan and *C. hongkongensis* populations from Shantou [19]. This suggests that the F4 population still retains relatively high genetic diversity.

### 4.2. Genetic Differentiation of the Breeding Generations of C. ariakensis

The genetic distances based on the *cox1* gene fragments decreased from F0 to F4, suggesting that the generations became increasingly genetically uniform as a result of the selection process. This pattern aligned with the directional changes in allele frequencies resulting from selective breeding [65]. Genetic differentiation coefficients based on *cox1* gene sequences revealed significantly moderate genetic differentiation between selectively bred populations (F1 and F4) and the F0 population, while the F1 population showed extreme genetic differentiation from the F2, F3, and F4 populations [70], suggesting intense selection pressure during the breeding process [71]. However, intra-population genetic variation exceeded the inter-population variation regardless of whether the analysis was based on the *cox1* gene or *rrnL*, indicating that substantial genetic diversity persists within populations despite multiple generations of selective breeding, and complete genetic differentiation among generations has not occurred. This outcome may be attributed to localized selection effects, sampling bias, or limitations of the genetic markers used [72,73,74]. These findings suggest that the bred generations retain considerable potential and can provide abundant genetic material for further selection, while increased selection intensity could help stabilize the target traits and achieve breeding objectives.

## 5. Conclusions

Our study found that the mitochondrial *cox1* and *rrnL* genes of *C. ariakensis* exhibit high AT-content, with *cox1* displaying higher polymorphism than *rrnL*. Under successive selective breeding, genetic diversity indices based on *cox1* markedly declined, confirming that high-intensity artificial selection significantly impacted the populations and drove genetic background convergence. Nevertheless, genetic variation remained predominant within populations, retaining substantial diversity. These findings suggest that intensifying selection pressure could further solidify desirable traits, although fewer parental individuals used could result in an insufficient number of haplotypes. In conclusion, this work provides a molecular basis for germplasm breeding and adaptive restoration in *C. ariakensis*, and informs the development of sustainable breeding strategies that balance yield with genetic health. Based on these findings, we conclude that significant potential remains for the genetic improvement of *C. ariakensis*. To fully exploit this potential, we recommend intensifying selection pressure and expanding the breeding population in future work. This strategy will establish a robust material foundation, necessary for high-resolution genetic studies with expanded datasets in the future.

## Figures and Tables

**Table 1:** Genetic diversities of the five *C. ariakensis* populations based on partial *cox1* gene sequences. *P*: number of polymorphic loci; *H*: number of haplotypes; *Pi*: nucleotide diversity index; *Hd*: haplotype diversity index; *K*: average number of nucleotide differences.

Population	Sample Size	Haplotype	*P*	*H*	*Hd*	*K*	*Pi*	
F0	35	Hap_1, Hap_2, Hap_3, Hap_4, Hap_5, Hap_6, Hap_7, Hap_8, Hap_9, Hap_10, Hap_11	10	11	0.714	1.20336	0.00185	
F1	33	Hap_1, Hap_6	2	2	0.504	1.00758	0.00155	
F2	30	Hap_1, Hap_6, Hap_12	3	3	0.349	0.60690	0.00094	
F3	28	Hap_1, Hap_6	2	2	0.198	0.39683	0.00061	
F4	32	Hap_1, Hap_6, Hap_12	3	3	0.123	0.18750	0.00029	
Total	158		11	12	0.431	0.76248	0.00117

**Table 2:** Genetic distances between populations (below the diagonal) and within populations (bold values), as well as pairwise *Fst* (above the diagonal) of the five populations of *C. ariakensis* based on partial *cox1* genes sequences.

Populations	F0	F1	F2	F3	F4
F0	**0.00186**	0.05533 *	−0.00195	0.00604	0.07211 *
F1	0.00181	**0.00156**	0.15309 **	0.19563 **	0.32533 **
F2	0.00140	0.00148	**0.00094**	−0.02356	0.02376
F3	0.00125	0.00136	0.00076	**0.00061**	0.01027
F4	0.00117	0.00138	0.00063	0.00046	**0.00029**

Note: Values marked with * were significantly different (*p* < 0.05), and marked with ** were extremely different (*p* < 0.01).

**Table 3:** Genetic distances between populations (below the diagonal) and within populations (bold values), as well as pairwise *Fst* (above the diagonal) of the five populations of *C. ariakensis* based on partial *rrnL* sequences.

Populations	F0	F1	F2	F3	F4
F0	**0.00012**	0.00000	−0.00354	−0.00765	0.05490
F1	0.00006	**0.00000**	0.00000	0.00000	0.07423
F2	0.00006	0.00000	**0.00000**	0.00000	0.06667
F3	0.00006	0.00000	0.00007	**0.00000**	0.05849
F4	0.00026	0.00020	0.00020	0.00020	**0.00037**

**Table 4:** Results of hierarchical AMOVA based on the partial *cox1* sequences from the five *C. ariakensis* populations.

Source of Variation	Degrees of Freedom	Sum of Squares	Variance Components	Percentage of Variation
Among populations	4	6.213	0.0381	9.8046%
Within populations	153	53.642	0.3506	90.1953%
Total	157	59.854	0.3887	100%
Overall fixation index	0.0981			

**Table 5:** Results of hierarchical AMOVA based on the partial *rrnL* sequences of the five *C. ariakensis* populations.

Source of Variation	Degrees of Freedom	Sum of Squares	Variance Components	Percentage of Variation
Among populations	4	0.256	0.0013	5.0213%
Within populations	154	3.681	0.0239	94.9787%
Total	158	3.937	0.0252	100%
Overall fixation index	0.0502			

## Data Availability

Relevant information has been included in the article.
